# Healthy Intestinal Function Relies on Coordinated Enteric Nervous System, Immune System, and Epithelium Responses

**DOI:** 10.1080/19490976.2021.1916376

**Published:** 2021-04-30

**Authors:** Fatima B. Saldana-Morales, Dasom V. Kim, Ming-Ting Tsai, Gretchen E. Diehl

**Affiliations:** a Graduate School of Biomedical Sciences, Baylor College of Medicine, Houston TX USA; b Immunology Program of the Sloan Kettering Institute, Memorial Sloan Kettering Cancer Center, New York, NY USA; c Immunology and Microbial Pathogenesis Program, Weill Cornell Medical College, Cornell University, New York, NY, USA

**Keywords:** Enteric nervous systems, mucosal immunity, intestinal epithelium, mucosal immune response, intestinal microbiota

## Abstract

During both health and disease, a coordinated response between the epithelium, immune system, and enteric nervous system is required for proper intestinal function. While each system responds to a number of common stimuli, their coordinated responses support digestion as well as responses and recovery following injury or pathogenic infections. In this review, we discuss how individual responses to common signals work together to support these critical functions.

## Introduction

The primary role of the gastrointestinal (GI) tract is to digest and absorb nutrients and excrete waste products after digestion^1^. The small intestine is tasked with nutrient digestion and absorption^2^ and the large intestine with absorption of water, electrolytes, and water-soluble vitamins.^3^ Healthy function of the intestine is supported by multiple systems, including the epithelium, nervous system, and immune system.^4–6^ The epithelium forms a tight, flexible, and dynamic physical barrier that allows for nutrient absorption. The immune system promotes a hospitable environment for commensal microorganisms, defends against pathogens, and supports epithelial and neuronal functions including survival and repair. The enteric nervous system coordinates absorption, muscle control, and peristalsis and further supports immune and epithelial functions. In the steady state and after injury or infection, these systems sense common signals to promote digestion, support tissue growth, clear pathogens, and repair tissue damage.^7–9^ This review will highlight both the individual responses against common signals by the epithelial, immune, and neuronal compartments of the gut as well as outline how these signals converge to ensure proper tissue function.

## Intestinal Organization

Within the intestine, a single layer of tightly connected epithelial cells creates a physical barrier separating luminal contents from underlying tissues.^10^ Epithelial cells are connected through tight junctions, desmosomes, and adherens junctions.^10^ There are various types of intestinal epithelial cells (IECs) each with a specialized function. IEC are organized into two types of structures: villi and crypts.^11^ Villi, found only in the small intestine, protrude into the lumen thereby increasing surface area for absorption. Crypts, the home for intestinal stem cells, extend down toward the muscularis and are found in both small and large intestine.^12^

IECs have specialized functions. Enterocytes, the most common IECs, are responsible for nutrient and water absorption. Goblet cells secrete mucins, the glycoprotein constituent of mucus. Mucus creates a barrier limiting direct microbial interactions with the epithelium.^13^ Enteroendocrine cells secrete hormones such as glucagon-like peptide 1 and cholecystokinin to support digestion and metabolism.^14^ Paneth cells are limited to the small intestine and release growth factors that promote proliferation and differentiation of stem cells and antimicrobial peptides that shape the composition of the microbiota and limit microbial growth near the epithelium.^15^ M cells overlay organized immune structures including Peyer’s patches and isolated lymphoid follicles to transfer luminal antigens to underlying immune cells allowing for immune surveillance.^16^ M cells can also allow entry of pathogenic and nonpathogenic microorganisms into the tissue. Within the epithelium, intraepithelial lymphocytes (IELs) are also found. IELs are innate like T cells that can rapidly respond to pathogen infection and are critical to intestinal tolerance and epithelial barrier function.^17^

Below the epithelial layer is a loose connective tissue called the lamina propria (LP). The LP contains many cell types including various immune cells including dendritic cells (DCs), macrophages, and lymphocytes. These immune cells are essential for maintaining gut homeostasis, compartmentalization of the microbiota, and defense against pathogens by secreting cytokines or releasing cytotoxic proteins.^18–20^ The epithelial layer, LP and basement membrane together make up the mucosa.

Underneath the LP is the submucosa, a connective tissue layer containing blood vessels, nerves, and lymphatics. Specialized tissue-resident macrophages with anti-inflammatory properties associate with and promote survival of blood vessels and nerves by secreting growth factors such as bone morphogenetic protein-2 (BMP-2).^21^ In return, associated blood vessels and nerves secrete survival signals, such as colony-stimulating factor-1 (CSF-1) that support macrophage development.^21,22^ In the submucosa, a net of organized nerves form the submucosal plexus which regulates water and ion reabsorption. This plexus is one of the components of the intrinsic enteric nervous system (ENS).^23^

Below the submucosa is the muscularis externa which consists of two smooth muscle layers, a thin outer longitudinal layer that shortens and elongates the gut and a thicker inner circular layer of smooth muscle that causes constriction. The myenteric plexus, the second component of the intrinsic ENS, lies below the submucosa in between these two muscle layers where it controls GI movement.^23^ The microbiota induces signaling in the myenteric plexus and promotes the proliferation of enteric neuronal precursor cells.^24^ Somatosensory neurons in the myenteric plexus, including nociceptor containing neurons, coordinate responses between the submucosa and peripheral tissues. These somatosensory neurons are pseudounipolar cells with a single bidirectional axon and peripheral axon terminals that innervate skin, joints, and other peripheral tissue.^25^ Sensory neurons interpret the physiological state of the gut by recognizing stretch, nutrient absorption, and bacterial signals.^26^

The GI tract is constitutively exposed to exogenous substances including dietary components and intestinal microbes, and emerging data demonstrates that these signals are recognized by intestinal cells to help coordinate overall tissue function. The gut is continuously colonized with a diverse collection of microbes, collectively referred to as the microbiota. The microbiota consists of bacteria, viruses including bacteriophages, fungi, and parasites with the bacterial components being best characterized.^27^ Diet and evolution have shaped the bacterial communities found in the healthy gut which are dominated by Bacteroidetes and Firmicutes.^27,28^ While there is great variation between individual people, there is extensive functional overlap between microbes.^27^ There is also variation within the intestine with the colon having the highest bacterial load and microbial diversity.^29,30^

Healthy intestinal function relies on maintaining a balanced microbial community and the microbiota provides a number of critical functions. Intestinal bacteria extract energy from indigestible food components and synthesize essential nutrients such as vitamins K and .^27,31,32^ Additionally, intestinal microbes modulate host cells to support barrier function and defend against pathogens.^18,20,33^ In a mechanism referred to as colonization resistance, the microbiota prevents pathogen colonization of the intestine. The microbiota performs this function by limiting essential nutrients, producing microbial metabolites or anti-microbial products, and activation of immune mediated protection.^34^ As demonstrated in germfree mice, the microbiota is further critical for proper development of the intestine and gut immune system.^11,35–37^

**Figure 1.**
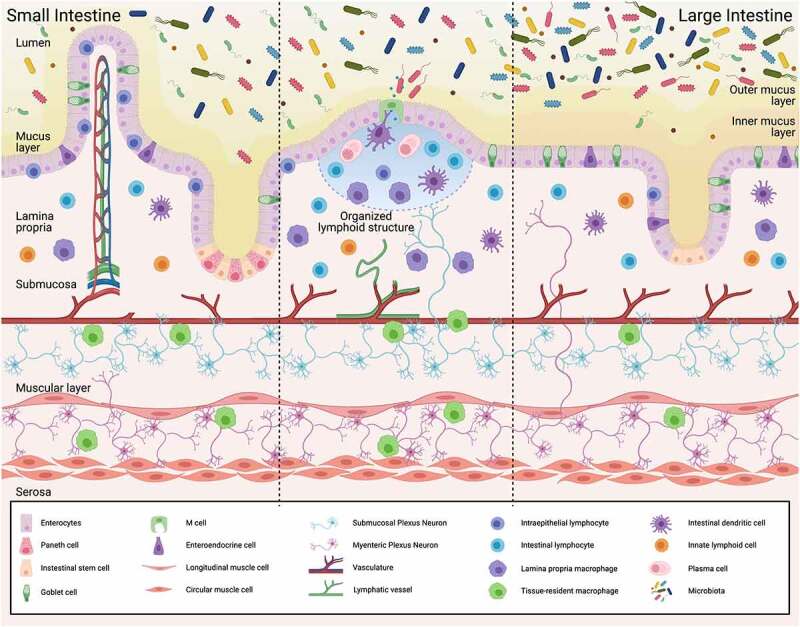
Intestinal organization promotes functional interactions between enteric nervous system, immune system, and epithelium to support intestinal functions. Created with BioRender.com

The structure of the gut allows for multiple opportunities for interaction between cellular components of the ENS, immune system and intestinal epithelium (Figure 1). This organized structure also allows for transduction of signals between the lumen and the different structural layers such as the submucosa or muscular layer.

## Role of intestinal microbiota in promoting GI functions

The initiation of bacteria-derived signaling relies on the recognition of numerous classes of molecules. The best-characterized signals consist of conserved bacterial structural components such as pathogen-associated molecular patterns (PAMPs) that include lipopolysaccharide (LPS) from the cell wall of gram-negative bacteria, lipoteichoic acid from gram-positive bacteria, peptidoglycan, and flagellin.^38^ Receptors responsible for recognizing PAMPs, known as pattern recognition receptors (PRRs), include membrane-bound Toll-like receptors (TLRs) and cytosolic nucleotide-binding oligomerization domain (NOD)-like receptors (NLRs).^38–40^ PRRs are expressed by IECs, immune cells, and enteric neurons and play critical roles in maintaining intestinal homeostasis by detecting microbe-derived signals.^40–43^ Due to the presence of the microbiota, the intestine is constantly exposed to a large number of microbial products and so these signals cannot be solely used to detect pathogenic organisms. How the intestine distinguishes between pathogenic and nonpathogenic organisms remains an active area of investigation.

IECs express low surface levels of TLRs and therefore have limited responsiveness to luminal microbial products.^40,41,44^ However, sensing of TLR ligands is crucial to support epithelial functions including tight junction protein expression, cell proliferation, and secretion of mucus and anti-microbial peptides by goblet and Paneth cells.^45,46^ Epithelial sensing of microbial products induces chemokines and cytokines which helps shape subsequent immune responses and supports intestinal barrier repair.^41,47^ In parallel, microbial recognition by the immune system leads to the secretion of cytokines and other factors that further supports barrier function and repair.^20^

Interestingly, and in contrast to other body sites, PRR activation in intestinal LP macrophages does not lead to secretion of proinflammatory cytokines and this is likely due to tolerance gained by constant exposure to bacterial signals.^48–50^ Intestinal macrophages and DCs in the gut secrete IL-10, TGF-β and other factors that limit inflammatory responses, including supporting the differentiation of Foxp3^+^ regulatory T (Treg) cells.^50,51^ In inflammatory conditions such as inflammatory bowel disease (IBD), intestinal macrophages become hyperresponsive to microbial signals and produce much of the inflammatory cytokines found in the tissue including TNFα^49^ and there is much ongoing work to understand this shift.

Microbial-derived signals are also important for normal ENS function. Mice deficient for TLR4, the main LPS receptor, or the TLR adapter protein myeloid differentiation primary response 88 (MyD88) have significant delays in gastrointestinal motility with reduced numbers of nitrergic (NO_2_ producing) neurons.^52^ This is likely a neuronal intrinsic defect as deletion of MyD88 in wnt-1 derived neurons leads to a similar phenotype.^52^ Further, in vitro incubation of enteric neuronal cells with LPS leads to NF-κB activation and increased cell survival.^52^ TLR2 is also expressed on enteric neurons and TLR2 deficient mice exhibit anomalies in ENS architecture with a reduced number of neurons in the myenteric ganglia resulting in intestinal dysmotility.^53^ TLR2 knockout mice are also deficient in smooth muscle glial cell line-derived neurotrophic factor (GDNF) that promotes neuronal survival and helps maintain ENS structure and function.^53^ Understanding how PAMPs regulate gastrointestinal motility will help to identify novel ways to utilize these signals to improve nutrient absorption.

Along with microbes themselves, microbe-produced metabolites play an essential role in modulating intestinal and systemic inflammation.^54,55^ These metabolites include intermediate and end products metabolized from host dietary sources, host molecules, and microbial products.^56^ There is accumulating evidence suggesting that microbial metabolites can be recognized by host receptors such as PRRs and G-protein coupled receptors (GPCR), and induce pathways affecting a range of host responses including inflammatory responses.^55^

Short-chain fatty acids (SCFAs), such as butyrate, acetate and propionate, are microbial metabolites that regulate epithelium, immune system and nervous system functions. SCFAs are recognized by common receptors including GPCR41 and GPCR43 as well as can also signal through unique receptors such as GPCR109a for butyrate and PSGR for acetate and propionate.^57–59^ SCFA are byproducts of fiber breakdown and are produced by obligate anaerobes Firmicutes, Bacteroidetes, and Clostridium.^59^ Acetate, by protecting IECs from apoptosis can protect from lethal infection with enterohaemorrhagic Escherichia coli O157:H7.^60^ The majority of butyrate, the primary energy source for colonic epithelial cells,^61^ is found in the colon lumen.^62,63^ Colonic epithelial cells metabolize butyrate through β-oxidation and the tricarboxylic acid pathway, consuming oxygen to favor anaerobic commensal bacteria over facultative anaerobic pathogens such as *Escherichia coli* and *Salmonella enterica* therefore preventing pathogen infection.^61,64^ This anaerobic environment stabilizes hypoxia inducible factor-1 (HIF-1), a transcription factor that regulates epithelial barrier function.^65,66^ In vitro, SCFA induce expression of tight junction and other barrier proteins, thereby increasing barrier function.^67^ Overall, the recognition of SCFAs by IECs maintains mucosal barrier function and protects the host against pathogen infection.

SCFAs have a number of anti-inflammatory effects on immune cells. Butyrate is a histone deacetylase (HDAC) inhibitor.^57–59^ In macrophages, butyrate decreases inflammatory cytokine production^57,68^ while also increasing microbial killing through increased phagocytosis and anti-microbial activity.^69^ SCFAs also increase differentiation and function of Treg cells, including IL-10 production^70–72^ which limits pathology in colitis models.^70,73^ HDAC inhibition can be counterbalanced by intestinal microbe metabolism of phytate to inositol-1,4,5-trisphosphate (InsP_3_) which also promotes epithelial growth and intestinal repair.^74^

SCFAs can also stimulate serotonin release by the sympathetic nervous system and influence central processes such as memory and learning.^75^ Butyrate, which can be recognized by the butyrate monocarboxylate transporter 2 in ENS neurons,^4^ significantly increases the proportion of choline acetyltransferase expressing enteric neurons and increases cholinergic-mediated colonic circular muscle contractions and intestinal motility.^76^ In isolated colon segments, butyrate can increase and propionate can decrease fecal propulsion velocity.^77^ Therefore, a balance of SCFAs in the lumen is necessary to achieve healthy gut motility.

Another group of bacterial metabolites able to modulate gut physiology are products of tryptophan metabolization. Tryptophan-derived metabolites including kynurenine, tryptamine, and indole regulate multiple intestinal functions through regulation of the aryl hydrocarbon receptor (AHR) which also recognizes xenobiotics.^78^ Mice deficient in AHR in IECs have impaired stem cell proliferation in colonic crypts,^77^ suggesting an epithelial intrinsic effect. AHR is also crucial for expanding Th17 cells and the production of IL-22 by Th17 cells and group 3 innate lymphoid cells (ILC3).^79,80^ IL-22 is essential for intestinal barrier function as it promotes epithelial cell proliferation and goblet cell secretion of mucus.^81^ Due to lost IL-22, AHR deficient mice are highly susceptible to pathogens such as *Citrobacter rodentium*, a model for enteropathogenic *Escherichia coli* (EPEC) and enterohaemorrhagic *E. coli* (EHEC).^80^ AHR activation induces cytochrome P450 (CYP1) enzymes to breakdown and clear AHR ligands. Overexpression of *Cyp1a1* in IECs leads to reduced Th17 cells and ILC3 indicating a key role for epithelial cells in producing AHR ligands to regulate immune responses.^82^ *Ahr* is also expressed in enteric neurons with the highest expression in the colon.^83^ This is relevant for gut motility because neuronal excitability genes such as *Kcnj12* are downstream of AHR activation and treatment with antibiotics reduces *Ahr* expression alongside delayed intestinal motility.^83^ This defect can be partially rescued by restoring neuronal *Ahr* expression, suggesting an important role for AHR in normal ENS function.^83^ Altogether, AHR is an important link between microbiota, diet and regulation of intestinal homeostasis.

Another important group of immune regulating metabolites is secondary bile acids. While the majority of bile acids are returned to the liver, a small fraction (approximately 5%) travel to the colon where they are converted into secondary bile acids by bacteria.^84,85^ Bile acids and their derivatives can modulate intestinal epithelium proliferation. While a primary bile acid, taurine-conjugated cholic acid, promotes the proliferation of IECs, its unconjugated secondary counterpart deoxycholic acid (DCA) inhibits proliferation in a farnesoid X receptor (FXR) dependent mechanism.^86^ In the LP, Lithocholic acid (LCA) derivatives 3-oxoLCA and isoalloLCA regulate T cell effector functions.^87,88^ 3-oxoLCA binds to the transcription factor RORγt and suppresses Th17 cell differentiation. IsoalloLCA promotes Treg cell differentiation by upregulating mitochondrial reactive oxygen species leading to increased Foxp3 expression.^87^ β- hydroxydeoxycholic acid (isoDCA) increases RORγt^+^ Tregs in an FXR dependent manner.^87,88^

Bile acids and secondary bile acids also modulate intestinal motility. Bile acids in the lumen of the small intestine slow transit to allow for efficient nutrient absorption while in the colon they stimulate motility.^89,90^ This is an example of the tight regulation the gut needs in order to function efficiently.

Human bile acid receptors, such as the secondary bile acid G-protein–coupled bile acid receptor 1 (GPBAR1, also known as TGR5), are highly expressed on enteric neurons.^91^ DCA, a TGR5 agonist, inhibits intestinal motility.^92^ There is evidence to suggest that bile acids can cause abdominal pain hypersensitivity by modulating FXR in mast cells to secrete nerve growth factors resulting in sensitization of TRPV1 channels in the dorsal root ganglia.^93^ Together, bile acid metabolites support proper physiological function of the gut through regulation of intestinal T cell differentiation, ENS activation, and gut motility.

Collectively, microbiota and byproducts of digestion signal through intestinal epithelium, immune cells, and enteric nervous system to support proper gut function. Sensing of microbes or microbial products fortifies barrier integrity, limits inflammation, and supports proper GI motility. More work needs to be done to define the intercellular networks and signaling between cells of each system to more clearly define how these individual signals are integrated.

## Pathogenic Infection

During infection or damage, the host needs to sense and understand pathogenic signals in order to orchestrate a proper response. Because of the large load of commensal microbes, the intestine must integrate numerous signals to distinguish between harmless and pathogenic microbes. Additional danger and damage signals, including pain, toxins, and microbial persistence, help amplify signals to support an effective immune response.

In contrast with commensals, pathogens have additional functional systems that help them colonize the host tissue. An example is the type III secretion system (T3SS) used by pathogenic Gram-negative bacteria, such as *Salmonella*, to deliver bacterial proteins directly into the cytoplasm of host cells.^38,94^ *Salmonella* has two T3SS which allow for epithelial cell uptake and intracellular survival in macrophages.^95^ Detection of the T3SS is a way for host cells to discriminate between pathogenic and commensal bacteria. T3SS are sensed by NLR family apoptosis inhibitory proteins (NAIPs) to activate NLRC4 inflammasome and induce secretion of IL-1β, leading to programmed cell death of macrophages.^95–98^ In parallel, intracellular flagellin detection via NLR neuronal apoptosis inhibitory protein 5 (NAIP5) and NAIP6 in the cytosol serves as a secondary signal to further promote the assembly of NLRC4 inflammasome and IL-1β production.^99,100^

Toxins produced by enteric pathogens are secreted factors that aid in the successful invasion of host cells and are an important etiology of pathogen-induced diarrheal diseases.^101^ Once a pathogen is attached to IECs, secreted toxins regulate water and electrolyte flux, form pores on target cells, regulate host cell protein synthesis, or affect the actin cytoskeleton to disrupt the intestinal epithelial barrier.^102^ For example, bacterial toxins from *Clostridium spp*. disrupt intestinal tight junctions by altering Rho GTPases and by interfering with actin ATPase activity.^103,104^ There are two main toxins produced by *Clostridium difficile*: TcdA and TcdB. Both inactivate Rho proteins and lead to increased intestinal permeability, disruption of chemotaxis, and cytoskeletal depolymerization.^105^ They can also disorganize F-actin and dissociate tight junction proteins occludin, ZO-1 and ZO-2.^106^ Similarly, *C. botulinum* C3 toxin disassembles actin filaments and disrupts tight junctions.^103^ By disrupting the host mucosal barrier, toxins facilitate pathogen colonization of the host intestine. Toxins can also induce protection. TcdA and TcdB also activate IEC apoptosis which limits the spread of *C. difficile* infection in vivo.^107^

The host immune system plays a key role in toxin-mediated defenses. After disruption of the gut epithelial barrier and induction of innate immune responses, *C. difficile* TcdA and TcdB inactivation of Rho GTPase leads to inflammasome activation and IL-1β production.^108^ TcdA and TcdB interfere with neuronal responses through inactivation of RhoGTPases. TcdB inhibits neurotransmitter release while TcdA causes release of substance P and calcitonin gene-related peptide (CGRP) which stimulate intestinal secretion as well as inflamamtion^109^. Substance P and CGRP promote intestinal macrophage release of pro-inflamamtory cytokines like TNFα.^109,110^

There are many other bacterial toxins that damage host systems and modulate their responses. IECs can detect very low levels of bacterial pore-forming toxins (PFT), including pneumolysin, alpha-hemolysin, streptolysin O, and aerolysin.^111^ Bacterial PFTs damage the intestinal epithelium and disrupt barrier integrity.^112,113^ Subcytolytic concentrations of these toxins phosphorylate p38 MAPK in IECs resulting in proinflammatory responses early during infection.^111^ PFTs also contribute to pathogen spread by suppressing immune responses including inhibiting neutrophil migration or lysing immune cells.^112,113^ Activation of the nervous system by bacterial toxins has been shown in the skin, where *Staphylococcus aureus* directly activates sensory neurons in the mouse via the toxins N-formyl peptides and alpha-hemolysin. These toxins upregulate sensory neuron release of CGRP, galanin, and somatostatin and suppress *S. aureus*-induced innate immune activation by phagocytes, which have the highest neuropeptide receptor expression.^25,114^ It will be important to identify if similar systems function during intestinal infections.

## Mechanical signals

While toxins and other microbial products can be recognized directly, cellular and tissue damage also activate warning systems and inflammatory responses. Stimuli such as stretch, heat, mechanical stress, pain, and cold are recognized by nociceptive sensory neurons as early signs of injury or infection and activate defense mechanisms.^115,116^ In addition to sensory neurons, nociceptive neuron activation can also release neuropeptides like substance P and CGRP, which will result in sensation of pain or burning.^117^

Stretch, an indirect indicator of digestion, growth, and gut distension, is recognized by nociceptors like the Piezo channel mechanoreceptor. In drosophila, Piezo channels regulate the differentiation of stem cells to enterochromaffin cells in the gut.^118^ Stretching and bending result in Piezo2 signaling in IEC cells leading to serotonin release which regulates gut functions such as motility.^119,120^ These data suggest that the development of a healthy epithelium requires mechanical and sensory signals. It remains unclear if mechanisms involved in gut development are used to restore a healthy barrier after infection. If so, these represent intriguing therapeutic targets.

In other mucosal sites like the lungs, mechanical responses like coughing can result in increased bacterial spread.^121,122^ *Mycobacterium tuberculosis* (Mtb) glycolipid sulfolipid-1 (SL-1) activates nociceptive neurons in vitro, and Mtb lacking SL-1 cannot stimulate a cough response in vivo.^114,123^ As above, it will be interesting to identify if similar signaling occurs in the intestine.

Nociceptive neurons’ role in the gut during bacterial infections has not been completely elucidated.^124^ LPS and other bacterial cell wall components can sensitize TRPV1 and TRPA1 nociceptors, resulting in pain hypersensitivity.^125,126^ In the skin, *S. aureus* causes pain by interacting with Nav1.8 nociceptor neurons in a TLR independent manner.^114^ Activation of GABA receptors and TLRs by bacterial components can alter pain sensitivity.^127^ Food antigens can also sensitize abdominal pain by inducing food antigen-specific IgE antibodies and mast cell-dependent histamine release.^128^ Nociceptor signaling is protective in some bacterial infections, such as *Salmonella*, where nocioceptor release of CGRP leads to reduced M cell density resulting in fewer entry points for *Salmonella*.^129^ Nocioceptor CGRP also increases colonization by commensal microbes that can further limit *Salmonella* infection.^129^

After the acute phase of infection or inflammation, the immune system releases soluble factors, such as resolvins and neuroprotectins, that indirectly desensitize nociceptive sensory neurons and reduce pain.^130,131^ In chronic conditions such as IBD, patients have increased expression of TRPV1 nociceptors alongside increased abdominal pain.^132–134^ However, the role of nociceptors in IBD is still unknown.

Substance P and CGRP receptors are also expressed by other cell types. Substance P is expressed by epithelial cells, several types of immune cells, and glia,^135^ while CGRP receptors are expressed by many immune cells. CGRP is released in response to TRPV1 activation during tissue damage and activates immune populations including T cells, B cells, macrophages, mast cells, and dendritic cells to enhance immunity by promoting inflammatory cytokine secretion.^136–138^ CGRP also promotes hematopoiesis as well as release of hematopoietic stem cells to the blood stream.^138^

Altogether, pain can be sensed by nociceptors in response to mechanical stretch and infections. The immune system regulates pain through pro- and anti-inflammatory mediators during steady state and disease. Similarly, pain acts as a danger signal during tissue damage. The cross-regulation between nociceptive neurons and the epithelium and immune system is used to maintain intestinal function as well as in defense after infection.

## Concluding Remarks

**Figure 2.**
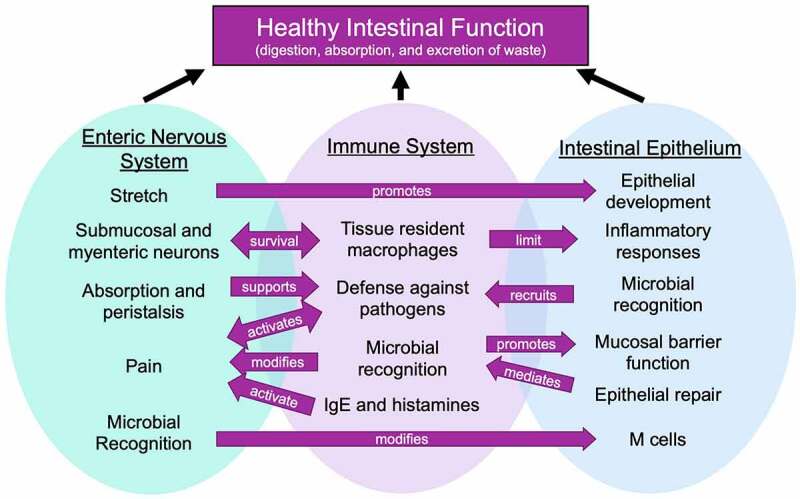
Coordinated sensing by the enteric nervous system, immune system and epithelium supports intestinal functions

A healthy and functional gut relies on proper responses to common signals in the intestine, including bacterial components, microbe-derived metabolites, and mechanical signals. Different systems within the gut work together to sustain barrier integrity and defend against pathogens, and these systems are essential to maintain balanced immunity and normal digestive and absorptive functions of the gut (Figure 2).

In this review, we provide examples of how the host intestinal epithelium, immune and neuronal systems recognize and respond to common signals to support healthy intestinal function (Table 1). We discuss how these different systems promote responses against normal microbiota and microbiota products, pathogens, and mechanical signals. The host intestinal systems induce regulatory and protective responses to commensal microbiota and defensive reactions to pathogens. Mechanical signals contribute to healthy intestinal functions or can enhance pathogen infection or clearance depending on the source and type of signal.

**Table 1.** Key Signals recognized by epithelial, immune and neuronal systems

Bacterial components			
Signals	Epithelial system	Immune system	Neuronal system
Microbial signals	Promote barrier integrity^20,41^ Secrete defense molecules^11,15,16,45^	Promote Tolerance^49,51^	Support neuronal survival^52,53^
**Metabolites**			
**Signals**	**Epithelial system**	**Immune system**	**Neuronal system**
SCFA	Energy source^61^ Create anaerobic niches^64^ Promote mucin secretion^45,57^ Promote Repair^74^	Anti-inflammatory response^57,58,68^ Promote Treg differentiation^70,72^	Influence memory and learning^75^ Mediate colonic motility ^76,^
AHR	Regulate epithelial proliferation^77^	Regulate Th17 and ILC3 functions^79,80,82^ Defense against pathogenic infection^80^	Regulate intestinal motility^83^
Bile acid and derivatives	Regulate epithelial proliferation^86^	Regulate T cell differentiation^87,88^	Mediate intestinal motility^89–92^
**Pathogenic infection**			
**Signals**	**Epithelial system**	**Immune system**	**Neuronal system**
T3SS	Barrier dysfunction^95^	Inflammasome activation^95,96^ Programmed cell death^95^	None Identified
Toxins	Barrier dysfunction^102,103,106,112,113^ Cell death^107,112^	Inflammasome activation^108^ Activate innate immune responses^108,112^ Inhibit immune responses^112,113^	Muscle contraction^109^ Neuropeptide release^25,114^ Inhibit immune response^25,114^
**Mechanical signals**			
**Signals**	**Epithelial system**	**Immune system**	**Neuronal system**
Physical signals	Neuropeptide release^119^	Indirectly aids in defense against pathogens^131^	Gut motility^120^
Chemical signals	Enhance barrier^129^	Release soluble factors to reduce pain^130,131^ Inflammatory responses^136,137^ Release pain-inducing substances^128^	Coughing^123^ Pain^114,125–128^

While many studies demonstrate how individual signals regulate one aspect of the intestinal system, the underlying signaling network and detailed mechanisms are less understood. Further, how these signals influence each other and converge into one functional outcome is only beginning to be addressed. The complexity of the signaling network as well as the plethora of activating and inhibitory signals make it challenging to study these interactions. Additionally, well-developed in vitro and in vivo systems are necessary to properly model the architecture of the gut required for the dissection of molecules and signaling pathways. Together, the coordinated response needs to be considered when attempting to manipulate intestinal function. Understanding the balance between these systems will allow us to design better therapeutics to target pathological states.

## Notes

National Institutes of Health

[AI125264].
